# Salicylic acid reduces MdPUB24-mediated ubiquitination of MdWRKY40 to suppress ethylene biosynthesis in apple fruit

**DOI:** 10.1093/hr/uhaf303

**Published:** 2025-10-29

**Authors:** Juntong Jin, Shijiao Lin, Weiting Liu, Aide Wang, Yinglin Ji

**Affiliations:** Key Laboratory of Fruit Postharvest Biology (Liaoning Province), College of Horticulture, Shenyang Agricultural University, Shenyang 110866, China; Hebei Key Laboratory of Horticultural Germplasm Excavation and Innovative Utilization, Hebei Higher Institute Application Technology Research and Development Center of Horticultural Plant Biological Breeding, College of Horticultural Science & Technology, Hebei Normal University of Science & Technology, Qinhuangdao 066004, China; Key Laboratory of Fruit Postharvest Biology (Liaoning Province), College of Horticulture, Shenyang Agricultural University, Shenyang 110866, China; Key Laboratory of Fruit Postharvest Biology (Liaoning Province), College of Horticulture, Shenyang Agricultural University, Shenyang 110866, China; Key Laboratory of Fruit Postharvest Biology (Liaoning Province), College of Horticulture, Shenyang Agricultural University, Shenyang 110866, China; Key Laboratory of Fruit Postharvest Biology (Liaoning Province), College of Horticulture, Shenyang Agricultural University, Shenyang 110866, China; Key Laboratory of Healthy Food Nutrition and Innovative Manufacturing (Liaoning Province), College of Food, Shenyang Agricultural University, Shenyang 110866, China

## Abstract

The plant hormone salicylic acid (SA) effectively suppresses ethylene biosynthesis in apple (*Malus domestica*) fruit. However, the underlying molecular mechanism remains unclear. Here, we identified a WRKY transcription factor, *MdWRKY40*, which was upregulated in response to SA treatment. MdWRKY40 functioned as a transcriptional repressor of the ethylene biosynthesis gene *MdACS1* (1*-aminocyclopropane-1-carboxylic acid synthase 1*). In addition, we found that the expression of U-box-type E3 ubiquitin ligase *MdPUB24* was downregulated following SA treatment. MdPUB24 interacted with MdWRKY40 and mediated its ubiquitination, leading to the degradation of MdWRKY40 via the 26S proteasome pathway, which was suppressed by SA. Together, these results suggest that the MdPUB24*-*MdWRKY40*-MdACS1* regulatory module mediates SA-induced suppression of ethylene biosynthesis by post-translational modification during apple fruit ripening. These findings offer new insights into the molecular basis of fruit ripening inhibition and shelf-life extension.

## Introduction

Ethylene plays a crucial role in regulating the ripening of climacteric fruits such as apple (*Malus domestica*) [1], tomato (*Solanum lycopersicum*) [2], pear (*Pyrus ussuriensis*) [3], and banana (*Musa acuminata*) [4]. ACS and ACO are recognized as key rate-limiting enzymes in ethylene biosynthesis [5, 6]. Silencing of *ACS* significantly reduces ethylene production and delays fruit ripening [7]. Inhibition of ACO enzymatic activity has been shown to suppress ethylene biosynthesis significantly [8]. Therefore, ethylene production could be controlled by regulating ethylene biosynthetic genes.

Regulation of ethylene biosynthesis has been widely studied in fruit [9–11]. It has been reported that other plant hormones regulate ethylene biosynthesis and fruit ripening, such as brassinosteroids [12], gibberellin [13], auxin [14], and melatonin (MT) [15]. Salicylic acid (SA), a ubiquitous plant-derived phenolic compound, is recognized as a kind of plant hormone and signaling molecule involved in plant responses to biotic and abiotic stresses [16–19]. Emerging evidence indicates that SA effectively suppresses ethylene biosynthesis across a range of horticultural crops. For example, acetylsalicylic acid treatment has been shown to inhibit the enzymatic activities of ACS and ACO, thereby suppressing biosynthesis of ethylene in kiwifruit (*Actinidia chinensis*) [20]. In pear (*Pyrus pyrifolia*), SA decreases the expression of *PpACS1a* and *PpACO1*, delaying fruit ripening [21, 22]. In apple, SA treatment significantly inhibited ethylene production, as well as the expression of *MdACS1* and *MdACO1* [23, 24]. Although many studies have documented the influence of SA on ethylene biosynthesis and fruit ripening, which only focused on enzyme activity and gene expression, the specific molecular mechanisms by which SA inhibits fruit ripening remain poorly understood.

Ethylene biosynthesis is known to be transcriptionally regulated in fruit. In apple, MdbHLH3 positively regulates *MdACS1* expression, thereby promoting ethylene biosynthesis during ripening [25], whereas MdERF2 represses *MdACS1* expression, resulting in reduced ethylene production [26]. MaWRKY49 and MaWRKY111 enhanced the transcription of *MaACS1* and *MaACO1*, accelerating banana fruit ripening [27]. In kiwifruit, AcWRKY40 upregulates the expression of *AcSAM2*, *AcACS1*, and *AcACS2* to promote ethylene biosynthesis [28]. Recent reports indicate that post-translational regulation, particularly via the ubiquitin proteolytic pathway, is important for ethylene-mediated fruit ripening. For example, the E3 ubiquitin ligase BRG3 reduces the ubiquitination of the repressor WRKY71, thereby delaying tomato ripening following hydrogen sulfide (H₂S) treatment [29]. In ripe bananas, MaBRG2 and MaBRG3 target and ubiquitinate MaMYB4. This relieves MaMYB4-mediated repression of *MaACS1*, *MaXTH5*, *MaPG3*, and *MaEXPA15*, ultimately inducing fruit ripening [30]. Although many studies have documented the role of the ubiquitin proteolytic pathway in ripening, the mechanisms by which this pathway influences apple fruit ripening remain largely undefined.

In this study, we demonstrate that exogenous SA treatment suppresses ethylene production and delays fruit ripening in apple. In contrast, inhibition of endogenous SA biosynthesis promotes ethylene production and accelerates ripening, indicating that SA functions as a negative regulator of ripening. SA enhances *MdWRKY40* expression while repressing *MdPUB24* expression, thereby suppressing the ubiquitination of MdWRKY40 by MdPUB24. Stabilized MdWRKY40 enhances its repression of *MdACS1*, thereby inhibiting ethylene biosynthesis. These findings elucidate a novel mechanism by which SA regulates apple fruit ripening and provide a theoretical foundation for strategies aimed at extending fruit shelf life.

## Results

### SA suppresses apple fruit ripening

**Figure 1:**
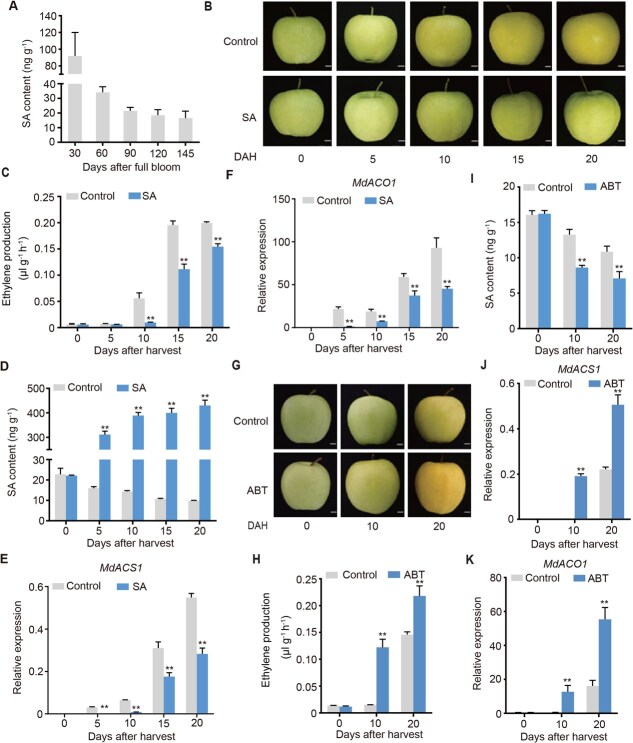
SA suppresses ethylene biosynthesis in apple fruit. (A) The level of endogenous SA was measured throughout the development of apple fruits in 2019. (B–F) Apple fruits harvested at commercial harvest were subjected to 2 mM SA treatment, followed by 20 days of storage at room temperature. Ethylene production (C), endogenous SA content (D), the expression level of *MdACS1* (E), and *MdACO1* (F) were measured during storage. (G–K) Apple fruits were subjected to treatment with a concentration of 2 mM of ABT and kept at room temperature for 20 days. Ethylene production (H), endogenous SA content (I), *MdACS1* expression (J), and *MdACO1* expression (K) were measured during storage. Scale bars, 1 cm. Control, fruits were not treated; SA, fruits were treated with SA; ABT, fruits were treated with ABT. Values represent means ± SD (*n* = 3 biological replicates). Student’s *t*-test assessed statistical significance (^**^P < 0.01, ^*^P < 0.05).

To investigate the effect of SA on ethylene biosynthesis in apple fruit, we determined endogenous SA levels. Our results revealed that endogenous SA content decreased significantly during early fruit development, with little change after 90 days after full bloom (DAFB) (Fig. 1A). In contrast, Lin *et al.* [13] showed that ethylene production increased during development [13], presenting an inverse trend to SA accumulation. Apples harvested at 145 DAFB (commercial harvest stage) were treated with 0.5, 1, and 2 mM SA. The results showed that 1 and 2 mM SA both significantly inhibited ethylene production (Fig. S1A). Therefore, we focused on the two effective concentrations (1 and 2 mM) next year. The results showed that both SA treatments suppressed the process of fruit yellowing and ethylene production compared with the control (Fig. 1B and C; Fig. S1B and C). SA treatment (2 mM) exhibited a more pronounced inhibitory effect. Therefore, 2 mM SA was selected for subsequent experiments.

Treatment with 2 mM SA increased endogenous SA content (Fig. 1D) and reduced the expression levels of the ethylene biosynthesis genes *MdACS1* (Fig. 1E) and *MdACO1* (Fig. 1F). In contrast, treatment with 1-aminobenzotriazole (ABT), an inhibitor of SA biosynthesis, accelerated fruit ripening and enhanced ethylene production (Fig. 1G and H). ABT treatment also reduced endogenous SA levels (Fig. 1I) and upregulated the expression of *MdACS1* (Fig. 1J) and *MdACO1* (Fig. 1K). Together, these findings support the role of endogenous SA as a negative regulator of ethylene biosynthesis during apple fruit ripening.

### MdWRKY40 is important for SA-reduced ethylene biosynthesis

**Figure 2:**
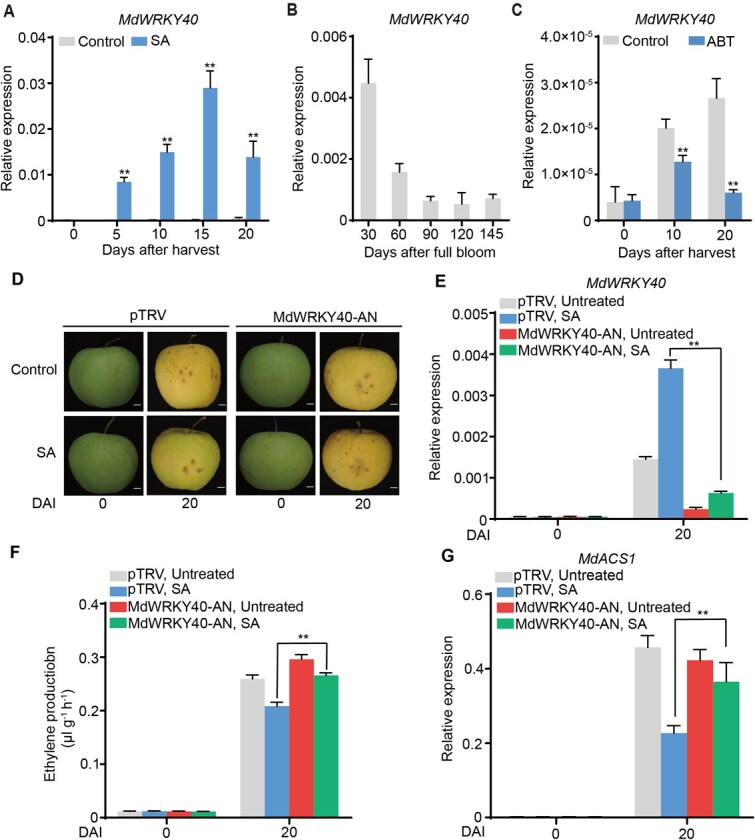
SA activates the expression of *MdWRKY40*. *MdWRKY40* expression after SA treatment during storage (A), *MdWRKY40* expression during apple fruit development (B), and *MdWRKY40* expression after ABT treatment during storage (C) were detected using RT-qPCR. (D–G) Silencing of *MdWRKY40* (MdWRKY40-AN) was achieved in apple fruits through a transient transformation mediated by *A. tumefaciens*. The injected fruits were treated with SA after 3 days. Subsequently, they were kept at room temperature for 20 days. (D) Apple fruit phenotype. Scale bars, 1 cm. *MdWRKY40* expression (E), ethylene production (F), and *MdACS1* expression (G) were measured. DAI, days after infiltration. Values represent means ± SD (*n* = 3 biological replicates). Student’s *t*-test assessed statistical significance (^**^P < 0.01).

To identify the transcription factors (TFs) significantly affected by SA during ripening, we mainly focused on those with a log2FC ≥ 3.0. And 16 TFs with a log2FC ≥ 3.0 were identified (Table S1) and detected the expression levels by standard polymerase chain reaction (PCR) (Fig. S2). We found that only *MdWRKY40* expression responded to SA treatment and consistent with the trend of ethylene during storage (Fig. S2). *MdWRKY40* expression was upregulated in response to SA treatment (Fig. 2A). Temporal expression profiling revealed that *MdWRKY40* expression decreased significantly during early fruit development with little change after 90 DAFB (Fig. 2B), which is consistent with ethylene production. This result indicates a positive correlation with SA accumulation and a negative correlation with ethylene production. Furthermore, *MdWRKY40* expression was reduced following treatment with ABT, an inhibitor of SA biosynthesis (Fig. 2C). Together, these findings suggest that MdWRKY40 is involved in mediating SA-induced suppression of ethylene biosynthesis.

To further investigate the function of MdWRKY40 in ethylene regulation, we examined its subcellular localization. The MdWRKY40-GFP protein localized to the nucleus (Fig. S3), consistent with its function as a transcription factor. We then transiently silenced *MdWRKY40* (MdWRKY40-AN) in apple fruit and treated the fruit with 2 mM SA 3 days later (Fig. 2D). We observed a significantly lower expression level of *MdWRKY40* in the MdWRKY40-AN fruit than in the control fruit (Fig. 2E). Following SA treatment, MdWRKY40-AN fruit exhibited significantly higher ethylene production than control fruit (Fig. 2F), as well as elevated expression of *MdACS1* (Fig. 2G). In contrast, *MdACO1* expression was not significantly altered between MdWRKY40-AN and control fruit (Fig. S4), suggesting that MdWRKY40 suppressed ethylene biosynthesis likely through transcriptional repression of *MdACS1* rather than *MdACO1*. In addition, *MdWRKY40* was transiently overexpressed in apple fruit (MdWRKY40-OE), followed by treatment with 200 mM ABT 3 days later (Fig. S5A). Compared with the control fruit, *MdWRKY40* expression was significantly upregulated in the MdWRKY40-OE fruit (Fig. S5B). After ABT treatment, both ethylene production (Fig. S5C) and *MdACS1* expression (Fig. S5D) were significantly lower than those in the control fruit. These results indicate that MdWRKY40 is involved in mediating SA-induced suppression of ethylene biosynthesis.

### SA-activated MdWRKY40 regulates the expression of the ethylene biosynthesis gene *MdACS1*

**Figure 3:**
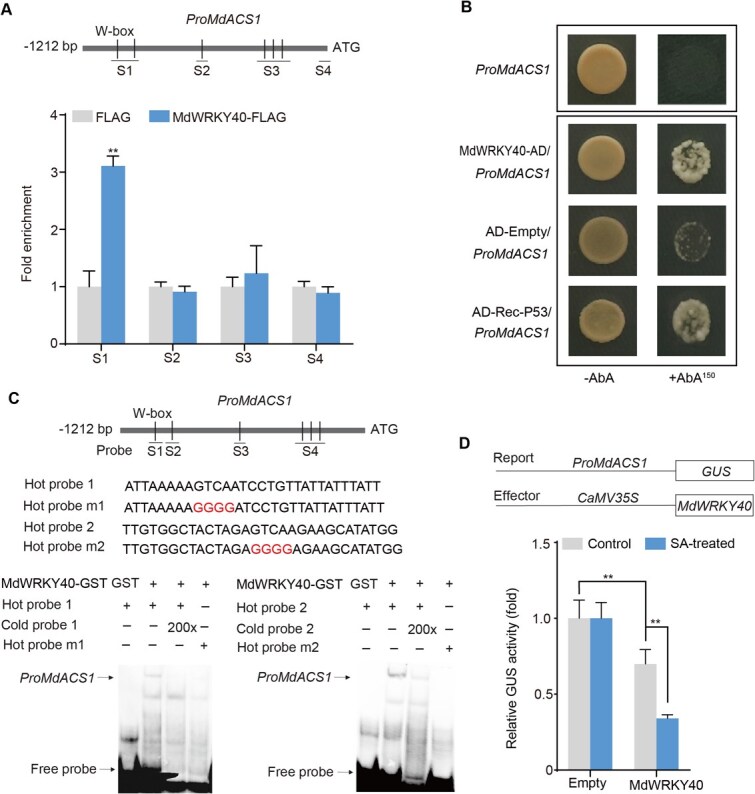
MdWRKY40 represses the expression of *MdACS1*. (A) ChIP-qPCR analysis confirmed the binding of MdWRKY40 to the *MdACS1* promoter *in vivo*. Four specific regions (S1–S4) of the *MdACS1* promoter were selected for analysis. This experiment was conducted three times. (B) The Y1H result showed that MdWRKY40 bound to the promoter of *MdACS1*. AbA (aureobasidin A) concentration was 150 ng ml^−1^. AD-Rec-P53/Pro*P53* was a positive control. AD-Empty/Pro*MdACS1* was a negative control. (C) EMSA result showed that MdWRKY40 interacted with the W-box in the *MdACS1* promoter. The recombinant protein MdWRKY40-GST was purified. The hot probe was biotin-labeled fragments (S1, S2), whereas the cold probe was an unlabeled competitor at a 200-fold concentration. Additionally, the mutant probe was a hot probe with four nucleotides altered. (D) The analysis of GUS activity indicated that MdWRKY40 downregulated *MdACS1*. Both *Pro35S::MdWRKY40* and *ProMdACS1::GUS* were introduced into the leaves of *N. benthamiana* to investigate GUS activity. The leaves were treated with SA for 3 h before being observed. Values represent means ± SD (*n* = 3 biological replicates). Student’s *t*-test assessed statistical significance (^**^P < 0.01).

To determine whether MdWRKY40 regulates the transcription of *MdACS1* or *MdACO1*, we analyzed the promoter regions of both genes and identified multiple W-box motifs (WRKY-binding sites). Chromatin immunoprecipitation (ChIP)-qPCR assays were then conducted to determine MdWRKY40 binding. The coding sequence (CDS) of *MdWRKY40*, tagged with FLAG, was overexpressed in apple callus. Results from ChIP-qPCR verified that the S1 fragment, which contains two W-boxes, was enriched in the presence of MdWRKY40-FLAG (Fig. 3A). In contrast, the *MdACO1* promoter exhibited no enrichment (Fig. S6), supporting the specific interaction of MdWRKY40 with the *MdACS1* promoter *in vivo*. This finding also explained that *MdACO1* expression was not changed in MdWRKY40-AN fruit. The yeast one-hybrid (Y1H) assay further confirmed that MdWRKY40 bound to the *MdACS1* promoter *in vitro* (Fig. 3B). To delineate the binding sites, an electrophoretic mobility shift assay (EMSA) was performed using four biotin-labeled fragments of the *MdACS1* promoter, which collectively contained six W-box motifs. MdWRKY40 bound specifically to the S1 and S2 fragments (Fig. 3C), whereas no binding was observed for S3 and S4 fragments (Fig. S7), corroborating the ChIP-qPCR results.

Subsequently, we explored how MdWRKY40 regulates the *MdACS1* promoter through a β-glucuronidase (GUS) reporter assay. The co-expression of *Pro35S*::*MdWRKY40* with *ProMdACS1*::*GUS* led to a marked decrease in GUS activity, an effect that was further intensified with SA treatment (Fig. 3D). Overall, these findings indicate that MdWRKY40, activated by SA, suppresses *MdACS1* transcription.

### MdPUB24 physically interacts with MdWRKY40

**Figure 4:**
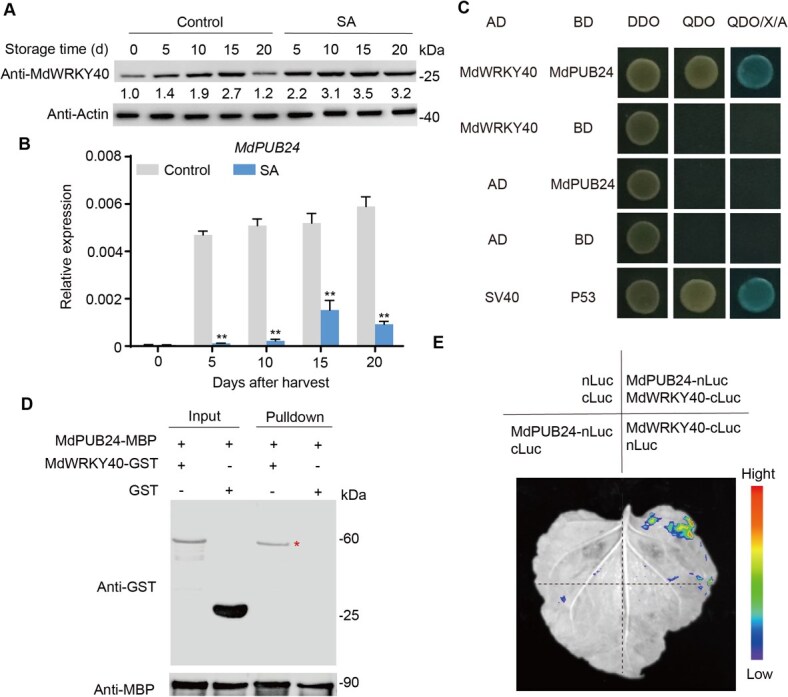
MdWRKY40 protein expression and interaction analysis with MdPUB24. (A) The MdWRKY40 protein level after SA treatment during storage was analyzed with an anti-MdWRKY40 antibody. Anti-actin antibody served as a reference to ensure consistent sample loading. (B) The expression levels of *MdPUB24* during fruit storage following SA treatment were assessed through RT-qPCR. Values represent means ± SD (*n* = 3 biological replicates). Student’s *t*-test assessed statistical significance (^**^P < 0.01). (C) The Y2H result showed that MdWRKY40 interacted with MdPUB24. SV40/P53 acted as positive controls, while AD/BD served as negative controls. (D) A pull-down assay was conducted to analyze the interaction between MdWRKY40 and MdPUB24. The recombinant proteins MdWRKY40-GST and MdPUB24-MBP were generated. Immunoblot analyses utilized GST and MBP antibodies. In the pull-down protein sample, the presence of a band identified by the GST antibody suggested that MdWRKY40 interacted with MdPUB24. (E) The LCI assay showed that MdWRKY40 interacted with MdPUB24. The nLuc and nLuc vectors were implemented as negative controls.

Previous studies have shown that several WRKY transcription factors are ubiquitinated and subsequently degraded via the 26S proteasome pathway during fruit ripening [29]. Based on this, we hypothesized that MdWRKY40 undergoes ubiquitination-mediated degradation during apple fruit ripening, a process modulated by SA treatment. To test this, we quantified MdWRKY40 protein levels in apple fruit with or without SA treatment. The result showed that the protein level of MdWRKY40 initially increased and subsequently decreased during ripening, whereas SA treatment significantly increased its accumulation (Fig. 4A), suggesting post-translational regulation of MdWRKY40.

Analysis of RNA-seq data identified an E3 ubiquitin ligase, *MdPUB24*, whose expression trend was consistent with ethylene production during storage and was downregulated in response to SA treatment (Fig. 4B; Table S2). To investigate the potential interaction between MdPUB24 and MdWRKY40, we first conducted a yeast two-hybrid (Y2H) assay, which revealed that MdPUB24 directly interacted with MdWRKY40 (Fig. 4C). This interaction was further validated by pull-down assays (Fig. 4D). To confirm the interaction in planta, a luciferase complementation imaging (LCI) assay was performed. Co-expression of MdPUB24-nLuc and MdWRKY40-cLuc resulted in a strong luminescence signal (Fig. 4E), providing further evidence of their physical interaction. Overall, these results reveal that MdPUB24 physically interacts with MdWRKY40, implicating a post-translational regulatory mechanism underlying SA-mediated modulation of ethylene biosynthesis.

### MdPUB24 mediates the ubiquitination of MdWRKY40, thereby inhibiting its transcriptional repressive activity

**Figure 5:**
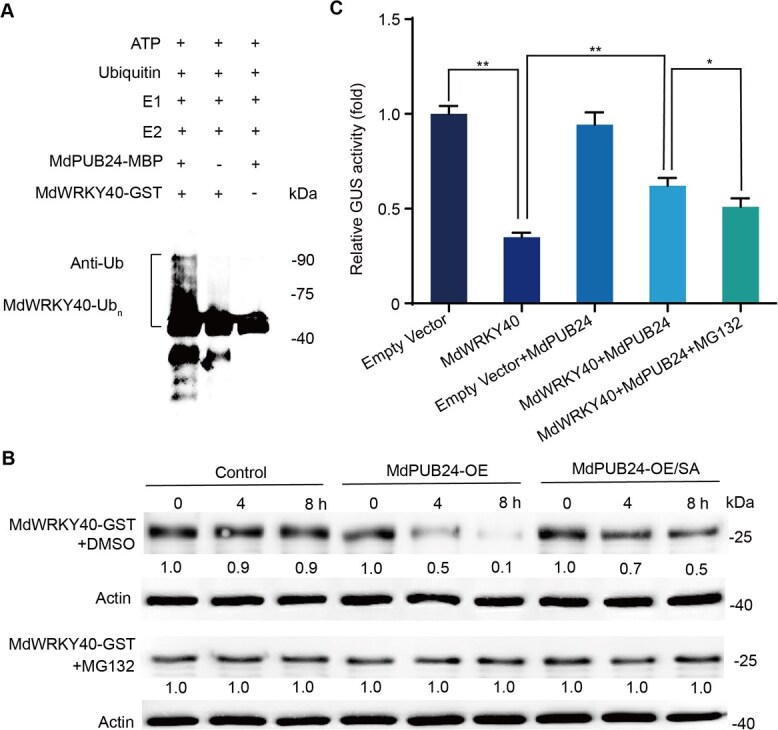
MdPUB24 mediates the ubiquitination of MdWRKY40, thus promoting its degradation and suppressing its transcriptional repression activity on the *MdACS1* promoter. (A) MdPUB24 mediates the ubiquitination of MdWRKY40 *in vitro*. MdWRKY40-GST was assayed for potential E3 ubiquitin ligase activity under conditions containing ubiquitin E1, E2, ATP, and MdPUB24-MBP. The abundance of MdWRKY40-GST protein was evaluated with an anti-ubiquitin antibody. (B) An *in vitro* cell-free degradation assay verified that MdWRKY40 degraded significantly faster in protein extracts from MdPUB24-overexpressing apple callus than in those from wild-type apple callus. The purified MdWRKY40-GST protein was added to total protein extracts and incubated for 4 and 8 h. The abundance of MdWRKY40-GST protein was assessed via an anti-GST antibody. (C) Analysis of GUS activity indicated that MdPUB24 inhibited the transcriptional repression activity of MdWRKY40 concerning the *MdACS1* promoter. Values represent means ± SD (*n* = 3 biological replicates). Student’s *t*-test assessed statistical significance (^**^P < 0.01, ^*^P < 0.05).

To investigate whether MdPUB24 influences MdWRKY40 stability through ubiquitination, an *in vitro* ubiquitination assay was performed. Recombinant MdWRKY40-GST and MdPUB24-MBP fusion proteins were incubated together at 30°C for 4 h with ubiquitin-activating enzyme E1, ubiquitin-conjugating enzyme E2, ubiquitin (Ub), and ATP. The result revealed the presence of high-molecular-mass bands corresponding to ubiquitinated MdWRKY40 upon co-incubation of MdWRKY40-GST and MdPUB24-MBP. These bands were absent when either MdPUB24-MBP or MdWRKY40-GST was omitted from the reaction (Fig. 5A), indicating that MdWRKY40 is directly ubiquitinated by MdPUB24. To further assess whether MdPUB24 regulates MdWRKY40 stability, a cell-free degradation assay was conducted. Total proteins extracted from control and MdPUB24 overexpression (MdPUB24-OE) callus were incubated with purified MdWRKY40-GST protein. The results showed that the MdWRKY40-GST protein was markedly degraded in the presence of MdPUB24-OE extracts, whereas SA treatment alleviated this degradation. In addition, this degradation was significantly inhibited by MG132, a 26S proteasome inhibitor (Fig. 5B). These results demonstrate that MdPUB24 promotes MdWRKY40 degradation through ubiquitination and the proteasome pathway.

To examine the functional consequence of MdWRKY40 ubiquitination on the transcriptional regulation of *MdACS1*, a GUS reporter assay was performed. Co-expression of *MdWRKY40* with the *MdACS1* promoter significantly suppressed GUS activity. However, co-expression with *MdPUB24* alleviated this suppression, resulting in increased GUS activity. Furthermore, MG132 treatment restored the repressive effect of MdWRKY40 on *MdACS1* expression (Fig. 5C). Collectively, these findings indicate that MdPUB24 mediates the proteasome-dependent degradation of MdWRKY40, thereby attenuating its transcriptional repression activity on *MdACS1*.

### MdPUB24 functions in SA-mediated repression of ethylene biosynthesis during the storage of apple fruits

**Figure 6:**
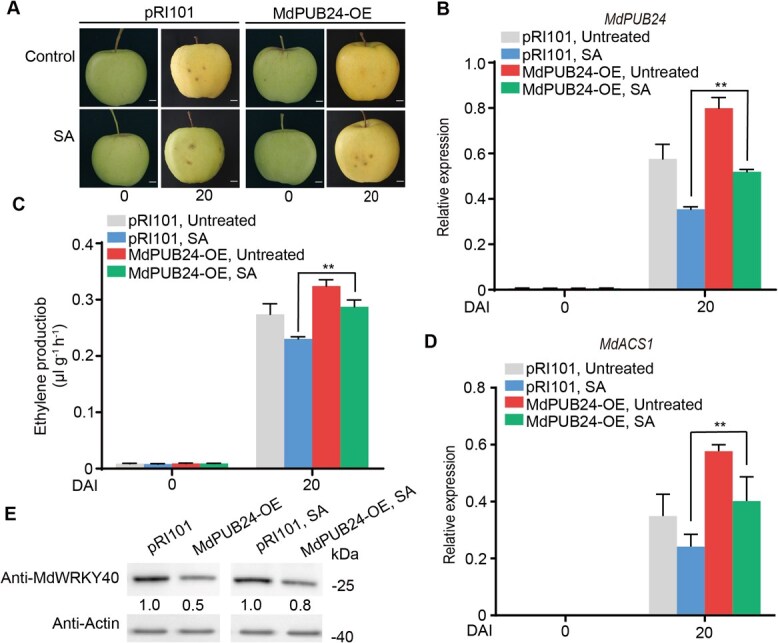
MdPUB24 is essential for the suppression of ethylene biosynthesis driven by SA. (A–E) *MdPUB24* was overexpressed in apple fruit (MdPUB24-OE) through transient transformation mediated by *A. tumefaciens*. The injected fruit was treated with SA after 3 days. They were subsequently kept at room temperature for 20 days. (A) Phenotype of the apple fruit. Scale bars, 1 cm. *MdPUB24* expression (B), ethylene production (C), *MdACS1* expression (D), and MdWRKY40 protein abundance (E) were investigated. Values represent means ± SD (*n* = 3 biological replicates). Student’s *t*-test assessed statistical significance (^**^P < 0.01).

To explore the MdPUB24 role in ethylene biosynthesis more deeply, the coding sequence of MdPUB24 was inserted into the pRI101 vector and subsequently delivered into apple fruit through *Agrobacterium*-mediated transformation to overexpress MdPUB24 (MdPUB24-OE). The fruits were treated with SA 3 days later (Fig. 6A). The expression of *MdPUB24* was significantly higher in MdPUB24-OE fruit compared to control fruit (Fig. 6B). Following SA treatment, both ethylene production (Fig. 6C) and *MdACS1* expression (Fig. 6D) were elevated in MdPUB24-OE fruit relative to controls, indicating that *MdPUB24* attenuates SA-mediated suppression of ethylene biosynthesis. Furthermore, we observed that the abundance of the MdWRKY40 protein was lower in MdPUB24-OE fruit than in control fruit (Fig. 6E), suggesting that MdPUB24 promotes the ubiquitination and subsequent degradation of MdWRKY40.

## Discussion

The regulation of fruit ripening is of considerable agronomic importance due to its impact on fruit quality, shelf life, and economic value. SA is a phytohormone ubiquitously distributed across the plant kingdom [16]. Numerous studies have demonstrated that exogenous SA treatment can delay fruit ripening in various species, including apple [23, 24], tomato [31], kiwifruit [20], and mango [32]. However, the precise role of exogenous SA in the regulation of ethylene biosynthesis remains incompletely understood.

Previous reports have indicated that endogenous SA levels vary during fruit development [33]. In apple, the content of endogenous SA progressively decreases as ripening advances [23], exhibiting an inverse trend relative to ethylene production. Exogenous SA has been shown to modulate the expression of SA biosynthesis enzymes and suppress ethylene production in apple [23]. In this study, we observed a gradual decline in endogenous SA levels during apple fruit ripening (Fig. 1A). Exogenous SA treatment not only elevated endogenous SA accumulation (Fig. 1D) but also significantly inhibited ethylene production (Fig. 1C) and the expression of key ethylene synthases *MdACS1* and *MdACO1* (Fig. 1E and F). In contrast, inhibition of SA biosynthesis using ABT reversed these effects. In *Arabidopsis thaliana*, SA is primarily synthesized via the phenylalanine ammonia-lyase (PAL) pathway [34]. Our transcriptomic analysis revealed that expression of the SA biosynthetic gene *MdPAL* was upregulated following SA treatment (Table S3), suggesting that exogenous SA may enhance endogenous SA accumulation by promoting *MdPAL* expression. Collectively, these findings indicate that exogenous SA stimulates endogenous SA biosynthesis, which in turn contributes to the suppression of ethylene production in apple fruit. This finding provided compelling evidence regarding the role of exogenous SA in ethylene biosynthesis in apple fruits.

Several studies have reported that SA and its derivatives modulate the expression and activity of ethylene biosynthesis-related genes, thereby reducing ethylene production [35]. For example, SA treatment decreases the expression of *OsACS1* and *OsACO1* in rice, resulting in suppressed ethylene production [36]. Similarly, treatment with ASA reduces both ethylene production and the activities of ACS and ACO during the early stages of kiwifruit ripening [20]. However, the molecular mechanisms underlying SA-mediated inhibition of ethylene biosynthesis in fruit have not been fully elucidated. In this study, we characterized the transcriptional and post-translational regulation of *MdWRKY40*, a WRKY transcription factor that mediates SA-induced suppression of ethylene biosynthesis in apple fruit. SA treatment led to a downregulation of *MdPUB24* (Fig. 4B), an E3 ubiquitin ligase that interacts with and ubiquitinates MdWRKY40 (Figs 4C–E and 5A), thereby targeting it for degradation via the 26S proteasome pathway (Fig. 5B). Reduction in MdPUB24 levels stabilized MdWRKY40, which in turn repressed the transcription of *MdACS1* (Fig. 5C) and attenuated ethylene biosynthesis. These findings delineate a mechanistic pathway through which SA modulates ethylene biosynthesis at both the transcriptional and post-translational levels.

In recent years, many plant U-box (PUB) protein families have been characterized for their biological functions. A large number of studies have revealed their key roles in plant resistance to biotic and abiotic stresses, as well as in growth and development. For example, AtPUB46, 48, and TaPUB1 are involved in plant response to drought stress [37, 38]. AtPUB44 participates in seed germination and early seedling growth [39]. Recently, U-box genes have been shown to regulate fruit ripening. It has been found that the *U-box 13*/*43*/*50*/*51* is highly expressed and predicted to have a role in the development and ripening of tomato fruit [40]. VlPUB38 negatively regulates fruit ripening by facilitating abscisic-aldehyde oxidase degradation in grapevine (*Vitis*) [41]. Here, the potential roles of U-box genes in apple fruit were evaluated. The results revealed that MdPUB24 negatively regulates the process of apple ripening through the ubiquitination and degradation of MdWRKY40 (Figs 4 and 5). This finding holds significant application potential for regulating fruit shelf life.

*MdACS1* and *MdACO1* encode key enzymes involved in ethylene biosynthesis. It reported that brassinosteroid-activated MdBZR1 represses *MdACS1* and *MdACO1* expression, thereby inhibiting ethylene production [12]. In our study, SA treatment significantly reduced the expression of *MdACS1* and *MdACO1* (Fig. 1E and F). However, SA-induced MdWRKY40 specially bound to the *MdACS1* promoter and suppressed its expression (Fig. 3A; Fig. S6). SA inhibited the expression of *MdACO1* independently of MdWRKY40. Previous reports showed that MdMADS5 only activated the expression of *MdACS1*, whereas MdCDPK7 directly phosphorylated MdACO1, thereby inducing its degradation and inhibiting ethylene biosynthesis [42]. MT prevented MdREM10 from promoting the transcription of *MdERF3*; in turn, MdERF3 reduced the transcriptional activation of *MdACS1*. On the other hand, MT also inhibited MdREM10-mediated promotion of *MdZF32* transcription, and MdZF32 further reduced the transcriptional activation of *MdACO1* [15]. These findings suggest that the regulation of *MdACO1* under SA treatment may involve transcriptional or post-transcriptional regulation, which warrants further investigation.

## Materials and methods

### Plant material and treatments

Apple (*M. domestica* cv. GD) fruits were harvested from the Liaoning Pomology Institute (Xiongyue, China) on the day of commercial harvest (145 DAFB). For SA treatment, the fruits were immersed in 0.5, 1, and 2 mM SA for a duration of 2 h. As a control, the fruits were also submerged in water for the same time. For ABT treatment, the fruits were immersed in 200 mM ABT for 2 h. All fruits were kept at room temperature for 20 days, with samples collected every 5 days.

The callus and *Nicotiana benthamiana* plants were grown in our laboratory as previously described [26].

### Measurement of ethylene production

The measurement of ethylene production in apples was conducted using a gas chromatograph (7890A, Agilent Technology, USA) following the methodology outlined by Li *et al.* [26]. An assay was performed utilizing five fruits from each group.

### RNA extraction and expression analysis

Total RNA extraction and cDNA synthesis were performed following the methodology outlined by Li *et al.* [26]. Real-time quantitative PCR (RT-qPCR) was utilized to assess gene expression levels. The conditions and procedures for the reactions were outlined by Li *et al.* [1]. The *Actin* gene from apple served as the internal control in this analysis.

### Measurement of SA content

The extraction and analysis of SA were conducted following the method outlined by Zhang *et al.* [43], with minor alterations. A 0.5-g sample of apple fruit was homogenized with 5 ml of 80% (v/v) methanol and incubated at 4°C for 16 h. After incubation, the mixture was centrifuged at 4°C for 10 min. Subsequently, the supernatant was subjected to vacuum drying to remove residual solvents. Finally, the dried extract was reconstituted with 1 ml of 10% (v/v) acetonitrile for high-performance liquid chromatography–tandem mass spectrometry (HPLC-MS/MS) (ZQ2000, Waters). A C18 solid-phase extraction cartridge purifying extract (ProElut; Dikma, China) was used.

### Subcellular localization

*MdWRKY40* CDS was inserted into the downstream of the GFP tag within the pRI101 vector to create the *Pro35S::GFP-MdWRKY40* construct. This construct was then co-infiltrated with a mCherry-labeled nuclear marker NF-YA4-mCherry [44] into the leaves of *N. benthamiana* for 3 days. As a control, an empty GFP was co-infiltrated with NF-YA4-mCherry. The localization of MdWRKY40 was observed by laser confocal microscope (TCS SP8, Leica).

### *Agrobacterium*-mediated infiltration

To silence *MdWRKY40*, a 1- to 300-bp partial CDS of *MdWRKY40* was cloned into the pTRV2 vector. To overexpress *MdPUB24*, its complete CDS was cloned into the pRI101 vector. Each recombinant construct was individually transformed into the *Agrobacterium tumefaciens* EHA105 strain. The infiltration of fruit was executed as outlined in a previous study [26]. Negative controls consisted of empty vectors. The injected fruits were treated with SA 3 days later.

### Y1H assay

*MdWRKY40* CDS was linked into the pGADT7 vector, while the promoter fragment of *MdACS1* was incorporated into the pAbAi vector. The Y1H experiment was conducted according to the method outlined in reference [26].

### Electrophoretic mobility shift assay

The MdWRKY40-GST recombinant construct was expressed in the *Escherichia coli* BL21 (DE3) (Transgen Biotech) competent cell and purified following previously described. The biotin-labeled promoter regions of *MdACS1*, which include the W-box motif, were synthesized by Sangon Biotech. EMSA assay was conducted according to the method of Kit (Beyotime Biotechnology).

### ChIP-qPCR assay

*MdWRKY40* was constructed into the pRI101-3×FLAG vector and transformed into *Agrobacterium* EHA105 strain. Apple callus was infected as previously described [26]. ChIP assay was performed according to the instruction of Kit (Cat. no. 56383; Cell Signaling Technology, USA). Flag antibody (Cat. no. YM3808; ImmunoWay, California, USA) was used to detect immunoprecipitation.

### GUS activation assay

*MdWRKY40* CDS was linked into the pRI101 vector to form the effector vector. Meanwhile, the promoter of *MdACS1* was cloned into the pCambia1300-GUS vector to form the reporter vector. Both vectors were co-infiltrated into the leaves of *N. benthamiana* through transient transformation mediated by *A. tumefaciens*. For SA treatment, a solution of SA (500 μM) was applied to the leaves of *N. benthamiana* 3 h prior to measurement. In the case of MG132 treatment, MG132 (50 μM) was infiltrated 12 h before measurement. The GUS activity assay was conducted as outlined in previous studies [26].

### Y2H assay

*MdWRKY40* and *MdPUB24* CDS were individually inserted into the pGADT7 and pGBKT7 vectors, respectively. The Y2H assay was conducted as described earlier [26].

### Pull-down assay

The cloning of MdPUB24 and MdWRKY40 CDS was carried out separately into the pMAL-C2X and pGEX4T-1 vectors, resulting in the formation of MdPUB24-MBP and MdWRKY40-GST constructs, which were subsequently transformed into BL21 (DE3) cells. A pull-down assay was conducted following the method outlined previously [45]. The proteins that were pulled down were analyzed with an anti-GST antibody (Cat. no. HT701-02; Transgen Biotech).

### Firefly LCI assay

The MdPUB24-nLuc and MdWRKY40-cLuc recombinant constructs were co-infiltrated into the leaves of *N. benthamiana* through transient transformation mediated by *A. tumefaciens* for 3 days, following the methods outlined earlier [46].

### *In vitro* ubiquitination assay

The proteins MdPUB24-MBP and MdWRKY40-GST were purified individually following the described above. An *in vitro* ubiquitination assay was conducted following an earlier method [47]. The proteins were resolved using SDS-PAGE gel. Detection of the MdWRKY40-GST protein was carried out using an anti-ubiquitin antibody (UBBiotech, Changchun, China).

### Cell-free degradation assay

The recombinant construct *Pro35S::MYC-MdPUB24* was transiently overexpressed into the leaves of *N. benthamiana*. As a negative control, an empty MYC vector was expressed. Total protein was extracted following a previously established method [42]. An anti-GST antibody was used to assess protein levels.

### Statistical analysis

Experiments were conducted three times, and results are presented as the mean ± standard deviation (SD). The statistical significance of the differences between the two datasets was evaluated using Student’s *t*-test (**P < 0.01; *P < 0.05). Data analysis was carried out with GraphPad prism software (GraphPad Prism 9.3).

## Supplementary Material

Web_Material_uhaf303

## Data Availability

The sequence information discussed in this article is accessible through the Genome Database for Rosaceae (https://www.rosaceae.org) or in the Genbank/EMBL libraries, identified by their accession numbers *MdWRKY40* (MD00G1143600), *MdPUB24* (MD12G1040800), *MdACS1* (U89156), *MdACO1* (AF030859), and *Actin* (EB13 6338).
