# Supplementary material for: Salicylic acid reduces MdPUB24-mediated ubiquitination of MdWRKY40 to suppress ethylene biosynthesis in apple fruit
Source: Hortic Res. 2025 Oct 29;13(2):uhaf303. doi: 10.1093/hr/uhaf303 (PMC12933665; doi:10.1093/hr/uhaf303)
Supplement: Web_Material_uhaf303 [file web_material_uhaf303.zip › Supplenmental Figures.pdf]

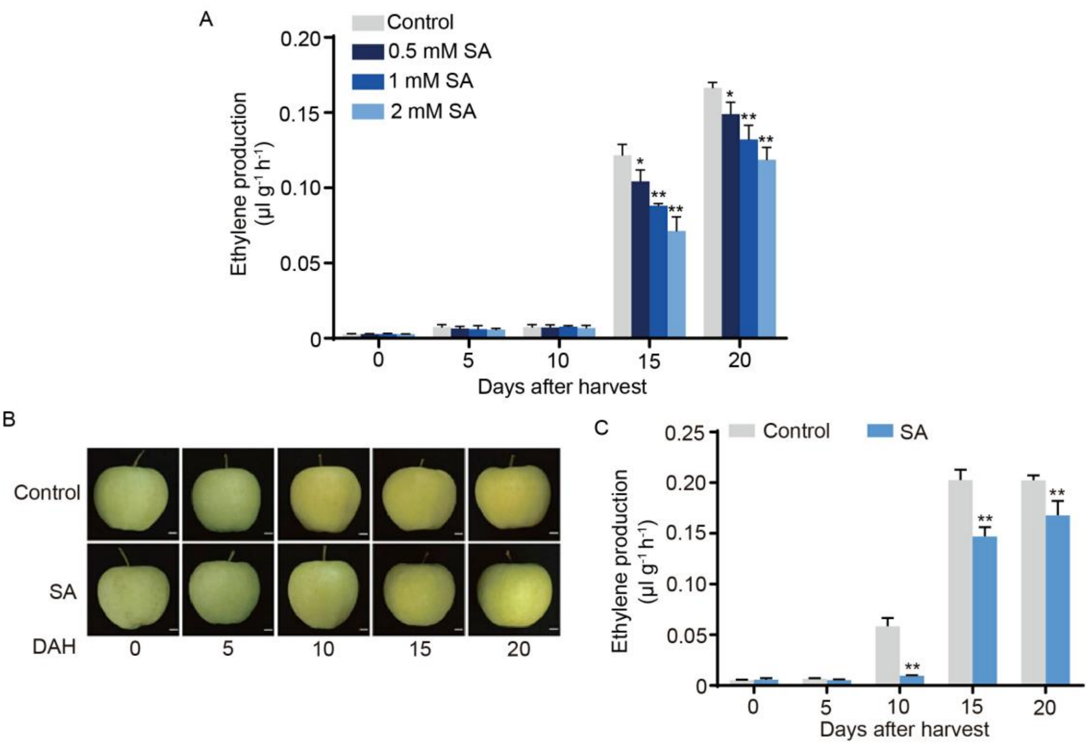

Figure S1. Salicylic acid (SA) suppresses ethylene production in apple fruit.

Apple fruits were harvested at commercial maturity, treated with SA, and subsequently kept at ambient temperature for 20 d. (A) Ethylene production of the apple fruit treated with 0.5, 1, 2 mM SA was assessed throughout the storage period in 2019. (B) Phenotype of the apple fruit treated with 1mM SA in 2020. Scale bar = 1 cm. (C) Ethylene production of the apple fruit treated with 1mM SA was assessed throughout the storage period in 2020. Values represent means ± SD (n = 3 biological replicates). Student's t-test assessed statistical significance(**P<0.01).

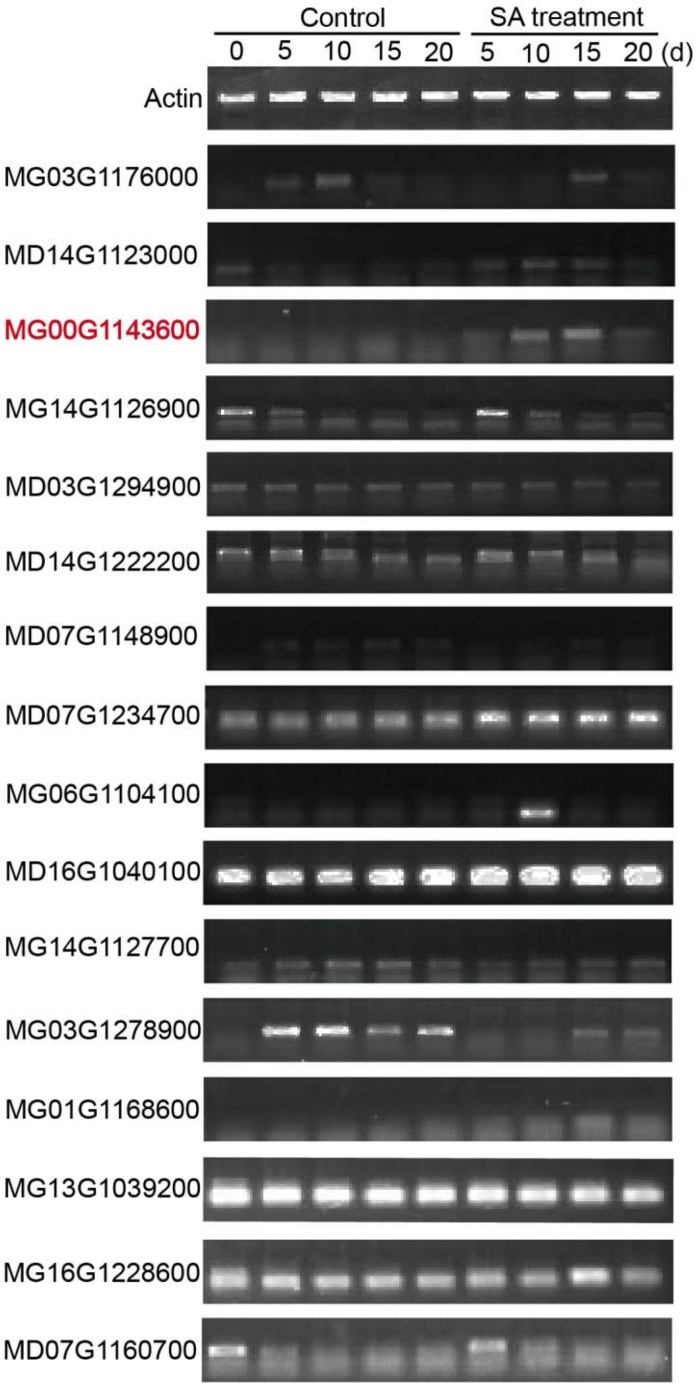

Figure S2. Differential gene expression after Salicylic acid (SA) treatment during storage. Apple fruits were treated with 2 mM SA for 2 h, followed by storage at room temperature for 20 d. Samples were collected at 5-day intervals during the storage period. Subsequently, the expression patterns of differentially expressed transcription factors identified via RNA sequencing were evaluated using standard PCR.

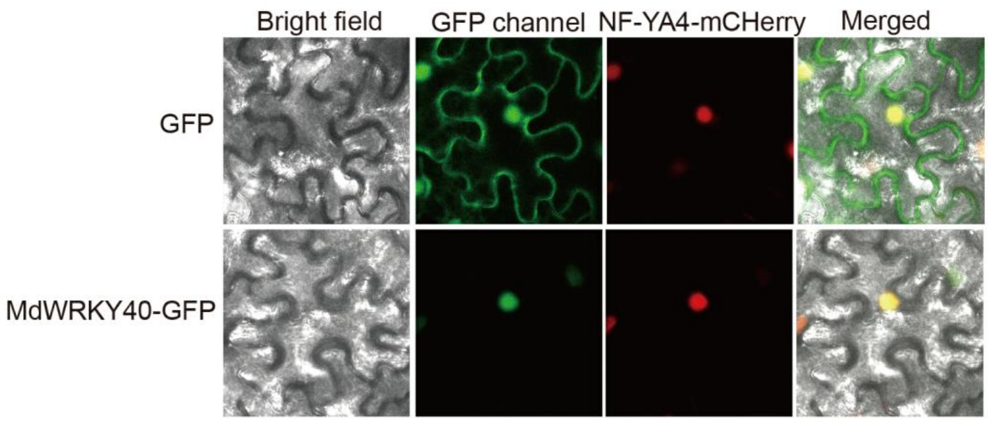

Figure S3. Subcellular localization of MdWRKY40.

The MdWRKY40-GFP recombinant construct was transiently expressed in the leaves of *Nicotiana benthamiana* for 3 d. An empty GFP vector served as the control in this experiment. NF-YA4-mCherry functioned as a marker for the nucleus. Scale bars, 50 μm.

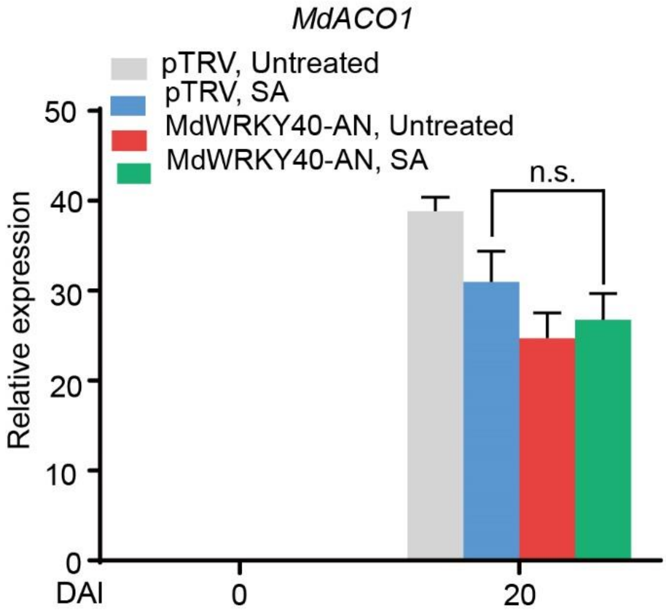

Figure S4. The expression level of *MdACO1* in *MdWRKY40*-silenced apple fruits. *MdWRKY40* was transiently silenced in apple fruits (MdWRKY40-AN), which were then treated with SA after 3 days of storage. The expression level of *MdACO1* was measured in MdWRKY40-AN apple fruits by RT-qPCR. DAI refers to days following infiltration. Values represent means ± SD (*n* = 3 biological replicates). Student's *t*-test assessed statistical significance (n.s, no significant difference).

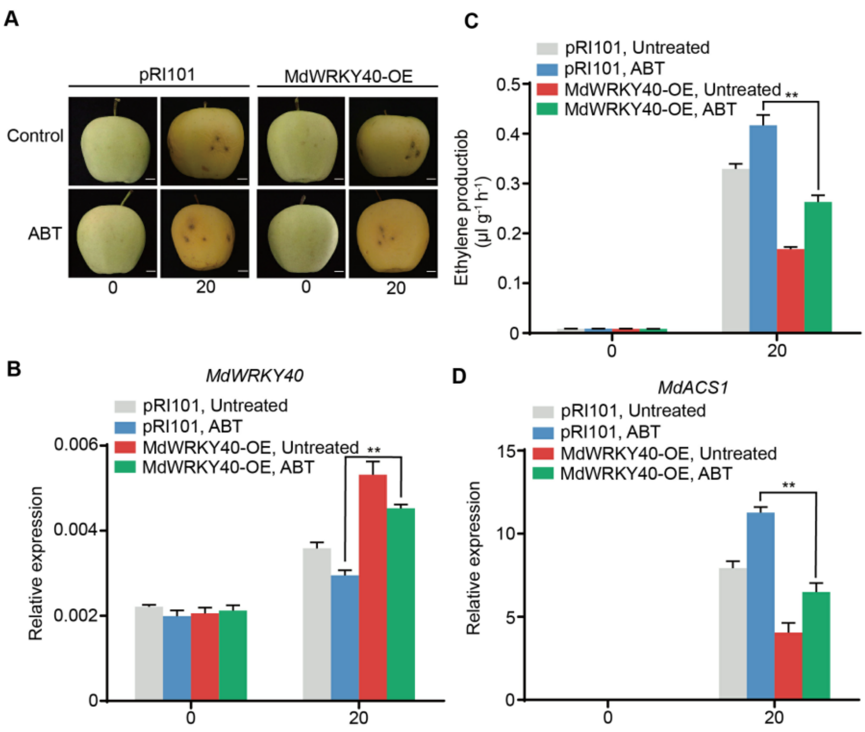

Figure S5. The function of MdWRKY4 in the Salicylic acid (SA)-mediated reduction of ethylene biosynthesis.

(A-D) Overexpression of *MdWRKY40* (MdWRKY40-OE) in apple fruits. The injected fruits were treated with SA after 3 d. Subsequently, they were kept at room temperature for 20 d. (A) Apple fruit phenotype. Scale bars, 1 cm. *MdWRKY40* expression (B), ethylene production (C), and *MdACS1* expression (D) were measured. DAI, days after infiltration. Values represent means ± SD (n = 3 biological replicates). Student's t-test assessed statistical significance (**P<0.01).

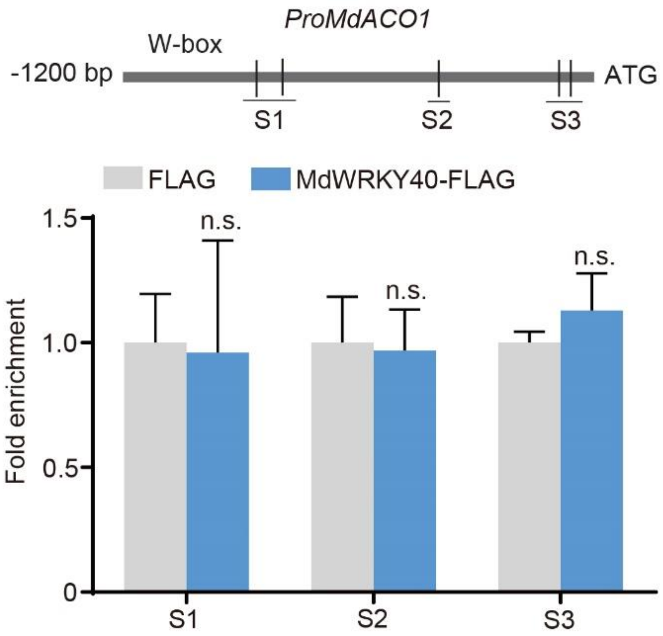

Figure S6. ChIP-qPCR verifies that MdWRKY40 does not interact with the *MdACO1* promoter. ChIP-qPCR was executed following Figure 3. We analyzed three specific areas of the *MdACO1* promoter. Values represent means ± SD (n = 3 biological replicates). Student's t-test assessed statistical significance (n.s, no significant difference).

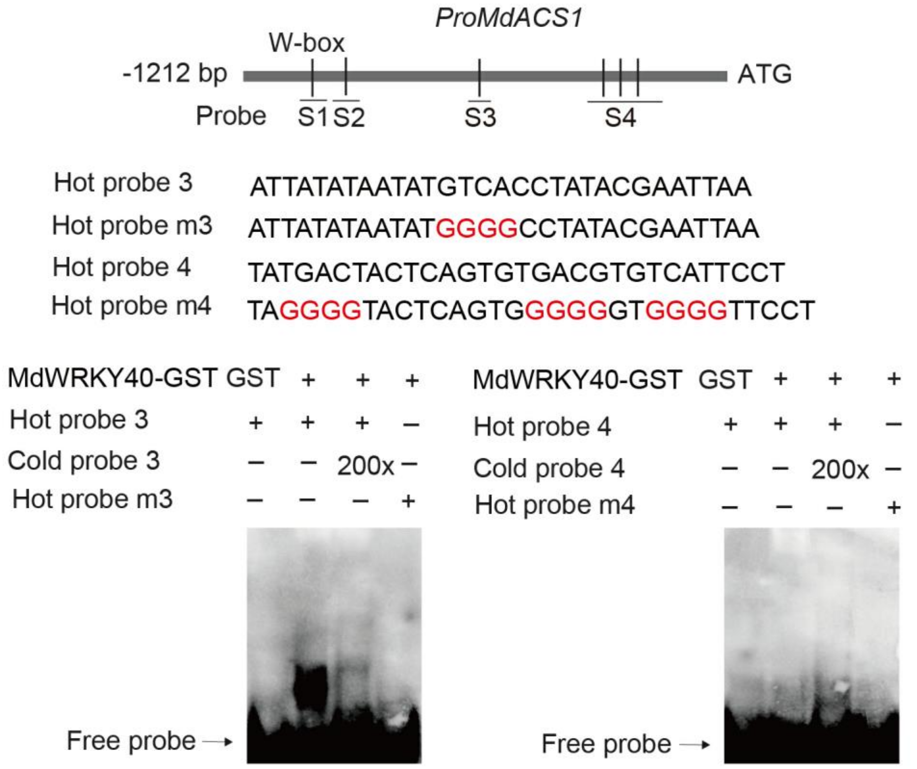

Figure S7. EMSA shows no binding of MdWRKY40 to the *MdACS1* promoter's S3 and S4 fragments.

EMSA assays revealed no binding of MdWRKY40 to the *MdACS1* promoter's S3 and S4 fragments. The hot probe was biotin-labeled S3, S4 fragments, whereas the cold probe was an unlabeled competitor at a 200-fold concentration. Additionally, a mutant probe was a labeled hot probe with four nucleotides altered.
